# Complement factor H attenuates TNF-α-induced inflammation by upregulating EIF3C in rheumatoid arthritis

**DOI:** 10.1186/s12967-023-04730-2

**Published:** 2023-11-23

**Authors:** Yimeng Jia, Bin Feng, Xin Ji, Xinping Tian, Lidan Zhao, Jiaxin Zhou, Wen Zhang, Mengtao Li, Yunyun Fei, Xunyao Wu

**Affiliations:** 1 Department of Rheumatology and Clinical Immunology, Chinese Academy of Medical Sciences, Peking Union Medical College Hospital, Peking Union Medical College, Beijing, China; 2 The Ministry of Education Key Laboratory, National Clinical Research Center for Dermatologic and Immunologic Diseases (NCRC-DID), Beijing, China; 3 Department of Orthopedic Surgery, Peking Union Medical College Hospital, Chinese Academy of Medical Science and Peking Union Medical College, Beijing, 100730 China; 4 Department of Nuclear Medicine, The First Affiliated Hospital of Nanjing Medical University, Jiangsu Province Hospital, Nanjing, China; 5 Department of Health and Medicine, Peking Union Medical College Hospital, Chinese Academy of Medical Sciences, Peking Union Medical College, Beijing, China; 6 Department of Medical Research Center, Peking Union Medical College Hospital, Chinese Academy of Medical Science and Peking Union Medical College, Beijing, China

**Keywords:** Complement factor H, Rheumatoid arthritis, Pyroptosis, Fibroblast-like synoviocytes, Eukaryotic translation initiation factor 3 subunit C

## Abstract

**Objective:**

To explore the role and underlying mechanism of Complement Factor H (CFH) in the peripheral and joint inflammation of RA patients.

**Methods:**

The levels of CFH in the serum and synovial fluid were determined by ELISA. The pyroptosis of monocytes was determined by western blotting and flow cytometry. The inflammation cytokine release was tested by ELISA. The cell migration and invasion ability of fibroblast-like synoviocytes (FLS) were tested by Wound healing Assay and transwell assay, respectively. The potential target of CFH was identified by RNA sequencing.

**Results:**

CFH levels were significantly elevated in the serum and synovial fluid from RA and associated with high sensitivity C-reactive protein (hs-CRP), erythrocyte sedimentation rate (ESR), and disease activity score 28 (DAS28). TNF-α could inhibit CFH expression, and CFH combined with TNF-α significantly decreased cell death, cleaved-caspase 3, gasdermin E N-terminal (GSDME-N), and inflammatory cytokines release (IL-1β and IL-6) of RA-derived monocytes. Stimulated with TNF-α increased CFH levels in RA FLS and CFH inhibits the migration, invasion, and TNF-α–induced production of inflammatory mediators, including proinflammatory cytokines (IL-6, IL-8) as well as matrix metalloproteinases (MMPs, MMP1 and MMP3) of RA FLSs. The RNA-seq results showed that CFH treatment induced upregulation of eukaryotic translation initiation factor 3 (EIF3C) in both RA monocytes and FLS. The migration of RA FLSs was promoted and the expressions of IL-6, IL-8, and MMP-3 were enhanced upon EIF3C knockdown under the stimulation of CFH combined with TNF-α.

**Conclusion:**

In conclusion, we have unfolded the anti-inflammatory roles of CFH in the peripheral and joints of RA, which might provide a potential therapeutic target for RA patients.

**Supplementary Information:**

The online version contains supplementary material available at 10.1186/s12967-023-04730-2.

## Introduction

Rheumatoid arthritis (RA) is a chronic autoimmune disease characterized by progressive synovitis that can lead to severe joint destruction and disability. TNF-α induced inflammation is a critical determinant for RA joint pathogenesis, and targeting TNF-α therapies has achieved significant improvements in RA treatment in recent years. However, a considerable proportion (approximately 40–44%) of patients fail to respond [1].

Recent studies have uncovered the important roles of the complement system in RA. Activated fragments and degradation products of complement are significantly elevated in the joint fluid and peripheral blood of RA patients [2, 3]. Mice deficient for C3aR, C5aR, or C6 led to decreased proximal joint IgG and C3 deposition in comparison to WT mice [4]. C4BP, one of the inhibitors of complement, has been found to have preventive and therapeutic value in collagen-induced arthritis (CIA) [5]. Our previous study has shown that complement C1q could promote monocyte pyroptosis and inflammatory cytokines release plus pentraxin 3 in RA [6].

Complement factor H (CFH) is an abundant soluble complement regulator essential for controlling the alternative pathway in blood and on cell surfaces, which protects cells and tissues from unintended complement-mediated injury [7]. CFH is secreted by cell types, including monocytes, fibroblasts, and endothelial cells, that likely contribute to local titers of the protein in tissues [8]. In collagen antibody induced- arthritis (CAIA) mouse models, complement receptor 2 combined with CFH effectively reduces the severity of joint swelling and cartilage damage [9]. In the present study, we have uncovered the role of CFH in inhibiting TNF-α-induced inflammation in monocytes and fibroblast-like synoviocytes (FLSs) and provided a potential strategy for promoting anti-TNF-α treatment in RA.

## Methods

### Patients and ethics

All newly-onset RA patients were enrolled in the Peking Union Medical College Hospital, and demographic information was demonstrated in Additional file 2: Table S1. Peripheral blood samples were collected from active RA (11 males and 44 females, aged 49.42±13.25 years) and healthy controls (5 males and 23 females, aged 36.43±11.61 years). Synovial specimens were collected from active RA (2 males and 15 females, aged 57.77±12.4 years) and OA patients (8 males and 6 females, aged 71.26±8.5 years) undergoing knee or hip arthroplasty (Additional file 2: Table S2). RA patients fulfilled the 2010 revised criteria of the ACR-EULAR classification [10].

### PBMC isolation and In vitro monocyte purification

Human peripheral blood mononuclear cells (PBMCs) were isolated with Ficoll-Paque density (DAKEWE, China) as previously described. CD14^+^ monocytes were purified using CD14 microbeads (130-107-576, Miltenyi Biotec) and maintained in RPMI 1640 supplemented with 10% fetal bovine serum (FBS) (Gibco, USA), 100 U/ml penicillin and 100 μg/ml streptomycin (15140122, ThermoFisher).

### FLS preparation

The synovial tissue was fully cut into pieces of 2–3 mm^2^ and enzymatically digested with 1 mg/ml type I collagenase (Sigma-Aldrich, St. Louis, MO, USA) in the shaker at 37 °C for 1 h. Following cell dissociation, the samples were filtered through a 70 μm cell strainer. Fibroblasts were pelleted by centrifugation at 1000 rpm for 10 min and plated in DMEM supplemented with 10% FBS (Gibco; Thermo Fisher Scientific, Waltham, MA, USA) and antibiotics (100 U/ml penicillin, 100 μg/ml streptomycin; Invitrogen, Carlsbad, CA, USA), and then put in a 37 °C, 5% CO2 incubator. The medium was changed after the cells adhered to the cell wall for 24 h. Once confluent, FLSs were trypsinized and diluted at a 1:3 split ratio for a new passage. The experiments were restricted to FLSs from passages 3–8.

### In vitro stimulation

For cytokines stimulation, freshly isolated monocytes and FLSs were incubated with 50 ng/ml TNF-α (Peprotech, 300-01A), IL-1β (Peprotech, 200-01B), and IL-6 (Peprotech, 200-06) for 24 h. The dose of CFH (R&D Systems, 4779-FH-050) used in our vitro studies was 5μg/ml.

### Cell viability assay

Cell viability was determined using a Cell Counting kit-8 (CCK-8 kit, Beyotime, China). 10% Cell Counting Kit-8 (CCK-8) reagent was added to each well and incubated for 30 min. The optical density was measured at 450 nm using a microplate reader (Bio-Rad, United States).

### Flow cytometry

Purified monocytes from each RA patient were seeded in 48-well culture plates, after treatment with TNF-α (50 ng/ml) or CFH (5 μg/ml) for 24 h. The cells were harvested, washed with cold PBS, and stained using an Annexin V–PE/7-AAD Apoptosis Detection Kit (BD PharMingen), according to the manufacturer’s instructions. Cells were then analyzed using a BD FACSAria™ II flow cytometer (BD Biosciences, San Jose, CA, USA).

### Wound healing assay

FLSs were seeded in 24-well plates at a density of 1×10^5^ cells/well. After the cells had adhered to the wall, they were scratched using pipette tips and then gently washed with PBS. Subsequently, the plate was put under a microscope to take pictures (0 h). After treatment with TNF-α (50ng/ml) or CFH (5 μg/ml) in the serum-free medium for 36 h, the scratched areas were photographed at the same magnification at the same position, and the migration area was analyzed.

### Transwell assay

FLS invasion was performed using the Boyden chamber method in 24-well plates with 6.5 mm diameter inserts containing 8 μm pores (Corning, NY, United States). The upper surface of the chambers was coated with Matrigel (50 μg/Well.BD Biosciences, Oxford, United Kingdom) which mimics the extracellular matrix. The FLSs (5×10^4^ cells in 200 μl) were suspended in a serum-deprived medium containing TNF-α (50 ng/ml), CFH (5 μg/ml), TNF-α+CFH (50 ng/ml and 5 μg/ml, respectively), and plated in the upper chamber. Simultaneously, a culture medium containing 10% FBS (600 μl) was placed in the lower chamber as a chemoattractant. Afterward, the system was incubated for 36 hours. Then FLSs were fixed with paraformaldehyde for 30 min and stained with 0.1% crystal violet for 20 min. The cells on the top surface of the membrane were scraped using a cotton swab, and then FLSs that migrated to the lower side were photographed under the microscope at 200 magnification. Five fields were randomly selected for cell counting using the ImageJ software, and the mean number of stained FLSs was calculated.

### Quantitative real-time PCR

After different treatments, total RNA was isolated from RA-FLS cells using TRIzol reagent (Invitrogen, USA). Then, total RNA was further reverse-transcribed into cDNA using the Prime Script RT Reagent Kit (Takara, China). The primers used for real-time PCR are listed in Additional file 2: Table S3. On the ABI-7500 Thermal Cycler (Applied Biosystems, USA), we used SYBR Green quantitative real-time polymerase chain reaction (qRT-PCR) (Takara, China) analysis to detect mRNA levels of every gene. Results were shown in the form of relative expression calculated by the 2^−ΔΔCT^ method.

### Enzyme-linked immunosorbent assay (ELISA)

Levels of CFH, IL-1β, IL-6, IL-8, MMP-1, and MMP-3 in the cell culture supernatant were measured with commercially available standard sandwich enzyme-linked kits.

### Western blotting

Proteins were separated by 10% SDS-PAGE and then transferred to a polyvinylidene fluoride (PVDF) membrane (Millipore, USA). The membranes were blocked with QuickBlock™ Blocking Buffer followed by incubation with primary antibodies, including anti-cleaved caspase 3 (9664S, Cell Signaling Technology), anti-GSDME (NBP2-80426, Novus Biological) and anti-EIF3C (NB100-511, Novus Biologicals), anti-β-actin (ab32503, Abcam) overnight at 4 °C. The membranes were washed three times and incubated with secondary anti-rabbit IgG (#7074, Cell Signaling Technology) or anti-mouse IgG (#7076, Cell Signaling Technology) for 1 h. Protein bands were visualized on the Western blotting detection system (Bio-Rad, USA)

### siRNAs transfection

Small interfering RNA (siRNA) against EIF3C and negative control siRNAs were synthesized by RiboBio (Guangzhou, China). The siRNA sequences are shown in Additional file 2: Table S3. Cells were cultured at 70–80% confluence and transfected with siRNAs using Lipofectamine 3000 reagent (Thermo Fisher Scientific, United States) following the manufacturer’s protocol.

### RNA sequencing and data analysis

For RNA sequencing, total RNA was extracted by the TRIzol method and quantified using a NanoDrop ND-1000 instrument. cDNA library construction for RNA transcriptome sequencing was performed by Beijing Novogene (Beijing, China), and volcano plots were performed for the differentially expressed genes (DEGs) using R 3.63 edge R software for statistical computing and graphics.

### Statistical analysis

Data are presented as mean ± standard error of measurement (SEM). The data were first performed with the normality distribution. Statistical analysis was performed using GraphPad Prism 8.0.1 software with one-way ANOVA. Two group comparisons were completed using a 2-tailed Student’s t-test or the Mann-Whitney test. The Pearson correlation analysis was adopted. The value of P < 0.05 was considered to be statistically significant.

## Results

### CFH is elevated in the serum and synovial fluid and correlated with disease activity in RA

**Fig. 1:**
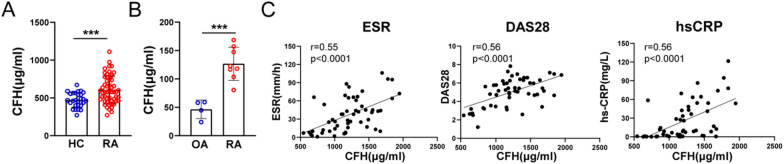
CFH is elevated in the serum and synovial fluid from RA. **A** CFH in serum from RA patients and healthy control detected by ELISA. (RA: n = 52; HC: n = 28). **B** CFH in synovial fluid from RA and OA patients detected by ELISA. (RA: n = 8; OA: n = 4). **C** Correlation analysis of CFH expression in serum of RA patients with ESR, DAS28, and hs-CRP respectively. Data are expressed as mean ± SEM. ***p < 0.001; RA: rheumatoid arthritis; OA: osteoarthritis; HC: healthy control; hs-CRP: high sensitivity C-reactive protein; ESR: erythrocyte sedimentation rate; DAS28: disease activity score 28

We first carried out ELISA to determine CFH levels in RA patients (Fig. 1A, B). We found that CFH was significantly higher in serum or synovial fluid of RA patients compared with those of healthy controls (606.6±185.8 μg/ml vs 472.4±96.7 μg/ml, p=0.0003) or osteoarthritis patients (126.8±29.2 μg/ml vs 46.5±16.3 μg/ml, p=0.0005). In addition, correlation analysis showed that CFH levels were positively correlated with ESR (r = 0.55, P < 0.0001), DAS28 (r = 0.56, P < 0.0001), and hs-CRP (r=0.56, P < 0.0001, Fig. 1C).

### CFH attenuates TNF-α–induced pyroptosis and inflammatory cytokines release of RA monocytes

**Fig. 2:**
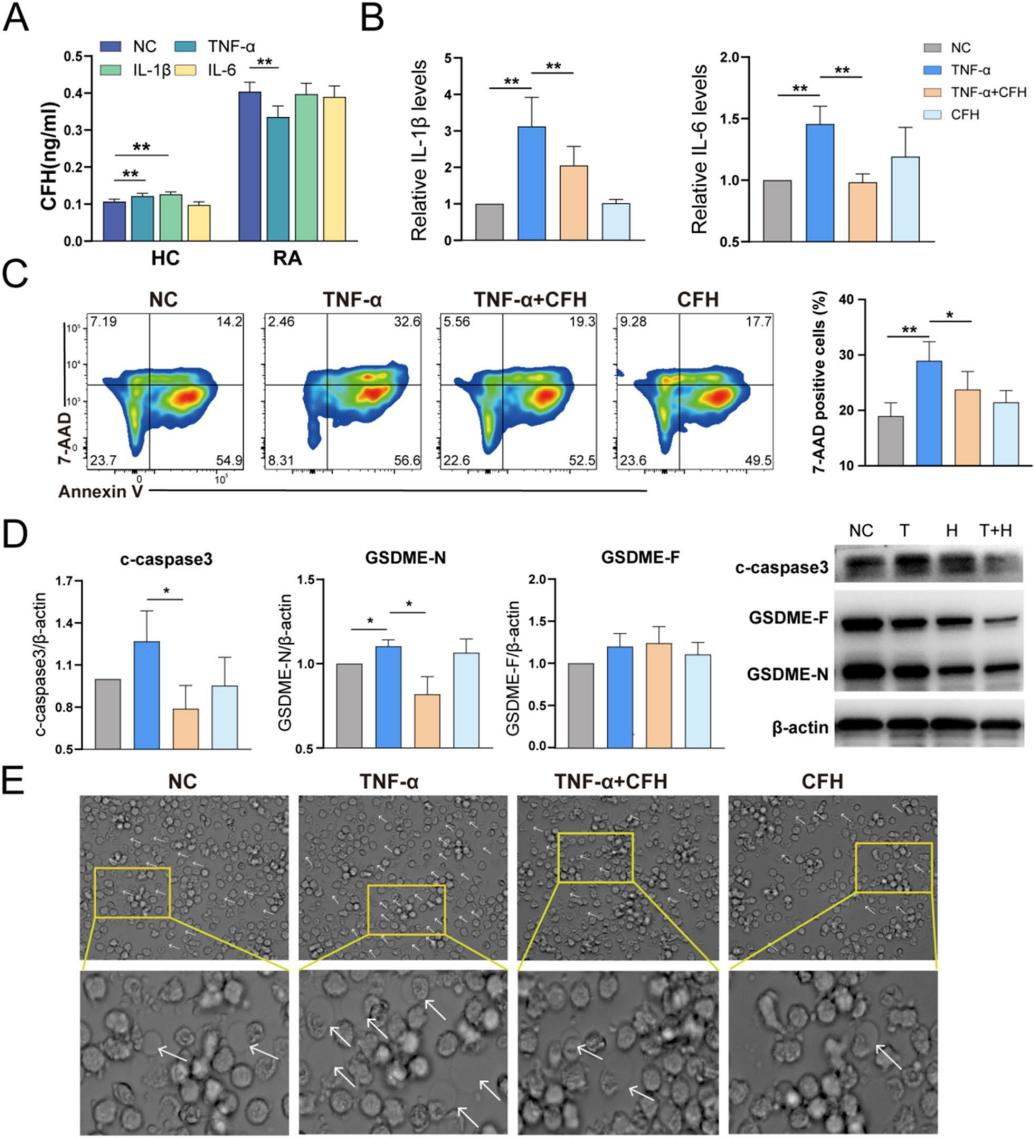
CFH attenuates TNF-α-induced pyroptosis and inflammatory cytokines release of RA monocytes. Purified CD14^+^ monocytes were pretreated with TNF-α(50 ng/ml), TNF-α(50 ng/ml) + CFH (5 μg/ml), or CFH (5 μg/ml) for 24 h (n = 6–9). **A** The expression of secreted CFH in the culture supernatant of RA patients and healthy control stimulated with TNF-α, IL-1β, and IL-6 detected by ELISA. **B** The expression of secreted cytokines (IL-1β, IL-6) in the culture supernatant of RA monocytes in each group was detected by ELISA. **C** Flow cytometric analysis of cells stained with annexin V/7-AAD to determine cell death and percentage of 7-AAD-positive cells. **D** The protein expression of c-caspase3, GSDME-N, and GSDME-F was measured by using Western blot. **E** Representative phase-contrast microscopy images of monocytes treated as indicated. Arrows indicate pyroptotic cell bubbles. Data are expressed as mean ± SEM. *p < 0.05; **p < 0.01; NC: negative control; T: TNF-α; T + H: TNF-α + CFH; H: CFH; IL-6: interleukin-6; IL-1β: interleukin-1β; c-caspase3: cleaved caspase-3; GSDME-N: gasdermin E N-terminal; GSDME-F: gasdermin E full length

We isolated monocytes and stimulated them with RA-related cytokines in vitro, including TNF-α, IL-1β, and IL-6, IL-18, IL-17A, GM-CSF, and IL-10 for 24 hours and detected CFH expression. TNF-αcould increase CFH levels in HC-derived monocytes but inhibit CFH expression in RA-derived monocytes (Fig. 2A and Additional file 2: Fig. S1A). Pro-inflammatory cytokines such as TNF, IL-6, and IL-1, are abundant in the synovium and synovial fluid in RA [11]. According to a 2019 scRNA-seq study, IL-6 is produced mainly by synovial fibroblasts, and macrophages are the main producers of IL-1 and TNF [12]. We therefore investigate the role of CFH in TNF-α-induced inflammation of RA-derived monocytes. As shown in Fig. 2B, CFH could significantly inhibit TNF-α-induced IL-1β and IL-6 release in RA-derived monocytes, indicating that CFH has an anti-inflammatory effect on RA.

A previous study has reported that TNF-α could induce pyroptosis in RA-derived monocytes by activating the caspase 3/GSDME pathway [13]. We therefore further investigated the effects of CFH on TNF-α-induced pyroptosis in RA-derived monocytes. Using flow cytometry, CFH+TNF-α decreased the percentage of 7-AAD-positive cells induced by TNF-α (Fig. 2C). Moreover, CFH combined with TNF-α significantly decreased c-Casp-3. TNF-α increased the expression of c-Casp-3, although there was no significant difference. TNF-α significantly increased the expression of GSDME-N, while CFH could inhibit TNF-induced GSDME-N expression. The expression of GSDME-F was not significantly different between groups (Fig. 2D). The typical morphologic changes characterized by cell swelling and large bubble blowing were also observed in each group (Fig. 2E). Taken together, these results indicate that CFH attenuates TNF-α–induced pyroptosis in peripheral blood monocytes from RA patients.

### CFH inhibits the migration, invasion, and TNF-α-induced expression of inflammatory mediators of RA FLSs

**Fig. 3:**
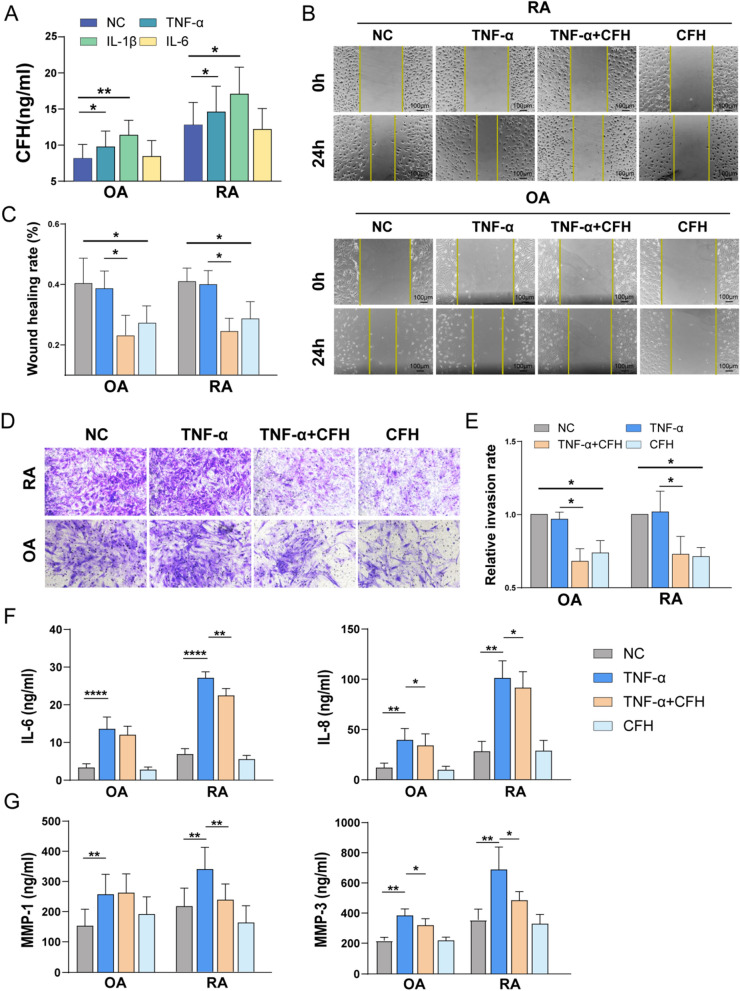
CFH inhibits the migration, invasion, and TNF-α-induced expression of inflammatory mediators of RA FLSs. The FLSs were pretreated with TNF-α（50 ng/ml), TNF-α（50 ng/ml) + CFH (5 μg/ml), or CFH (5 μg/ml) for 24 h (n = 5–10). **A** The expression of secreted CFH in the culture supernatant of RA-FLS and OA-FLS detected by ELISA. **B-C** The effect of CFH on cell migration was detected using wound healing assay. The scratching area was photographed at 0 h and 24 h. The scratch assay was presented as the percentage by which the original scratch area decreased at 24 h. Representative images (original magnification, × 200) are shown. **D-E** FLS invasion ability was measured by Transwell assay, and the invaded cells were photographed. The relative invasion rate was calculated by counting mean invaded cells from 5 randomly selected fields and then normalized to that in the NC group. Representative images (original magnification, × 200) are shown. **F–G** The expression of secreted cytokines (IL-6, IL-8) and MMPs (MMP-1, MMP-3) in culture supernatant of RA-FLS and OA-FLS in each group were detected by ELISA. Data are expressed as mean ± SEM. *p < 0.05; **p < 0.01; ***p < 0.001; ****p < 0.0001. MMP-1: matrix metalloproteinase-1; MMP-3: matrix metalloproteinase-3

We further investigate the effect of CFH on local inflammation in RA patients. We found that stimulation with TNF-α could increase the mRNA and protein levels of CFH both in RA and OA FLS (Fig. 3A and Additional file 2: Fig. S1B). Moreover, we found that CFH could significantly inhibit the migration and invasion ability of RA-FLS and OA-FLS (Fig. 3B–E). The cell-to-cell contact between RA fibroblasts and macrophages in the lining layer provokes IL-6 and IL-8 production and amplifies the inflammatory signaling cascades [14]. In accordance with what we observed in monocytes, CFH could also suppress TNF-α-induced proinflammatory cytokines (IL-6, IL-8). RA-FLS also secrete a variety of matrix metalloproteinases (MMPs) to drive joint destruction, among these, MMP-1 and MMP-3 can directly destroy type II collagen and thus promote cartilage destruction [15]. In particular, MMP-3 is a reliable marker of rheumatoid arthritis disease activity, imaging monitoring, prognosis, and response to treatment [16]. CFH could suppress TNF-α-induced production of MMP1 and MMP3 in the RA-FLS cells (Fig. 3F, G). However, CFH did not affect cell viability in both RA-FLS and OA-FLS (Additional file 2: Fig. S2) and exhibited only a mild effect on TNF-α induced inflammatory factors in OA-FLS (Fig. 3F, G). Taken together, these results support a protective role of CFH in RA, which could both inhibit the migration and invasion of FLS and attenuate TNF-induced inflammation in RA FLS and monocytes.

### EIF3C is a potential target for CFH to play a role in inhibiting FLS function

**Fig. 4:**
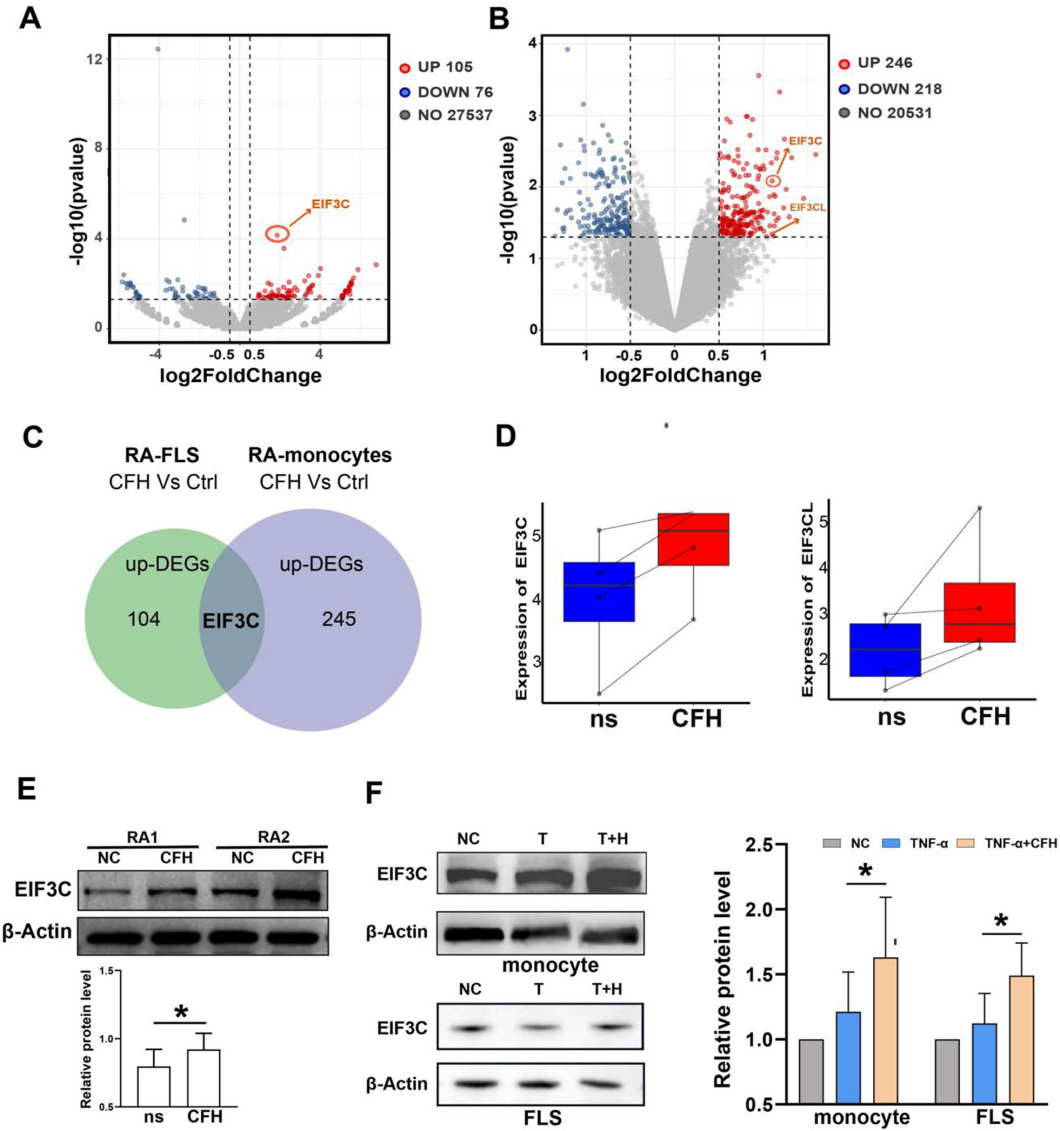
EIF3C is a potential target for CFH to play a role in inhibiting FLSs and monocyte function. Monocytes and FLS from RA patients were selected respectively, and cells from each patient were divided into two groups. One group was left untreated, and one group was treated with 5 μg/ml CFH for 6 h. **A** Volcano plot indicated up-regulated (red dots) and down-regulated (blue dots) genes (P value < 0.05 and |log2FC|> 0.5) by RNA sequencing in CFH-treated versus untreated RA monocytes. EIF3C is indicated. **B** Volcano plot indicated up-regulated (red dots) and down-regulated (blue dots) genes (P value < 0.05 and |log2FC|> 0.5) by RNA sequencing in CFH-treated versus untreated RA monocytes. EIF3C and EIF3CL are indicated. **C** We selected the genes upregulated in the CFH-treated monocytes and the genes upregulated in the CFH-treated FLS identified by RNA-sequencing and determined the intersecting proteins. We obtained one gene, namely EIF3C. DEGs: Differentially Expressed Genes. **D** The boxplot shows the relative expression of EIF3C and EIF3CL. **E** CFH upregulated the protein expression of EIF3C by using Western blot. **F** CFH combined with TNF-α upregulated protein expression of EIF3C in both RA FLSs and monocytes by using Western blot. Data are expressed as mean ± SEM (n = 6). *p < 0.05

To gain insights into the molecular mechanism of CFH, we performed RNA sequencing of CFH-treated and untreated FLSs from three RA patients. A total of 105 upregulated genes and 76 downregulated genes (with P value <0.05 and |log2FC| >0.5) were detected and visualized by volcano plot (Fig. 4A). CFH-treated and untreated monocytes were also used to carry out RNA sequencing from four RA patients. The volcano plot showed a total of 246 upregulated genes and 218 downregulated genes (with P value <0.05 and |log2FC| >0.5) (Fig. 4B). A detailed list of the differentially expressed genes is shown in Additional file 1. To identify the genes that play an important role in both RA-derived monocytes and FLSs, the upregulated genes related to the CFH treatment in monocytes and FLS were selected, and the intersecting proteins were identified. One gene was obtained, namely, EIF3C (eukaryotic translation initiation factor 3 subunit C) (Fig. 4C). We found both EIF3C and EIF3CL (eukaryotic translation initiation factor 3 subunit C like) were upregulated in the CFH-treated monocytes (Fig. 4D). The genetic sequence and encoded protein of EIF3CL are nearly identical to that of EIF3C. Moreover, EIF3C is the top 1 upregulated differentially expressed gene according to the P value in the CFH-treated FLS (Fig. 4A). Thus, we focused on EIF3C.

We used Western blotting to verify the reliability of RNA-seq. The results showed a significant increase in the EIF3C protein level in CFH-treated monocytes compared to the negative control (Fig. 4E). To further investigate whether the anti-inflammatory effect of CFH on TNF-α is also dependent on EIF3C, we assessed the effect of TNF-α and CFH on EIF3C expression in monocytes and FLS, respectively. The results are shown in Fig. 4F. The expression of EIF3C was significantly increased in both RA-derived monocytes and FLS with CFH and TNF-α combined stimulation.

### EIF3C knockdown upregulated TNF-α–induced expression of proinflammatory cytokines and MMPs

**Fig. 5:**
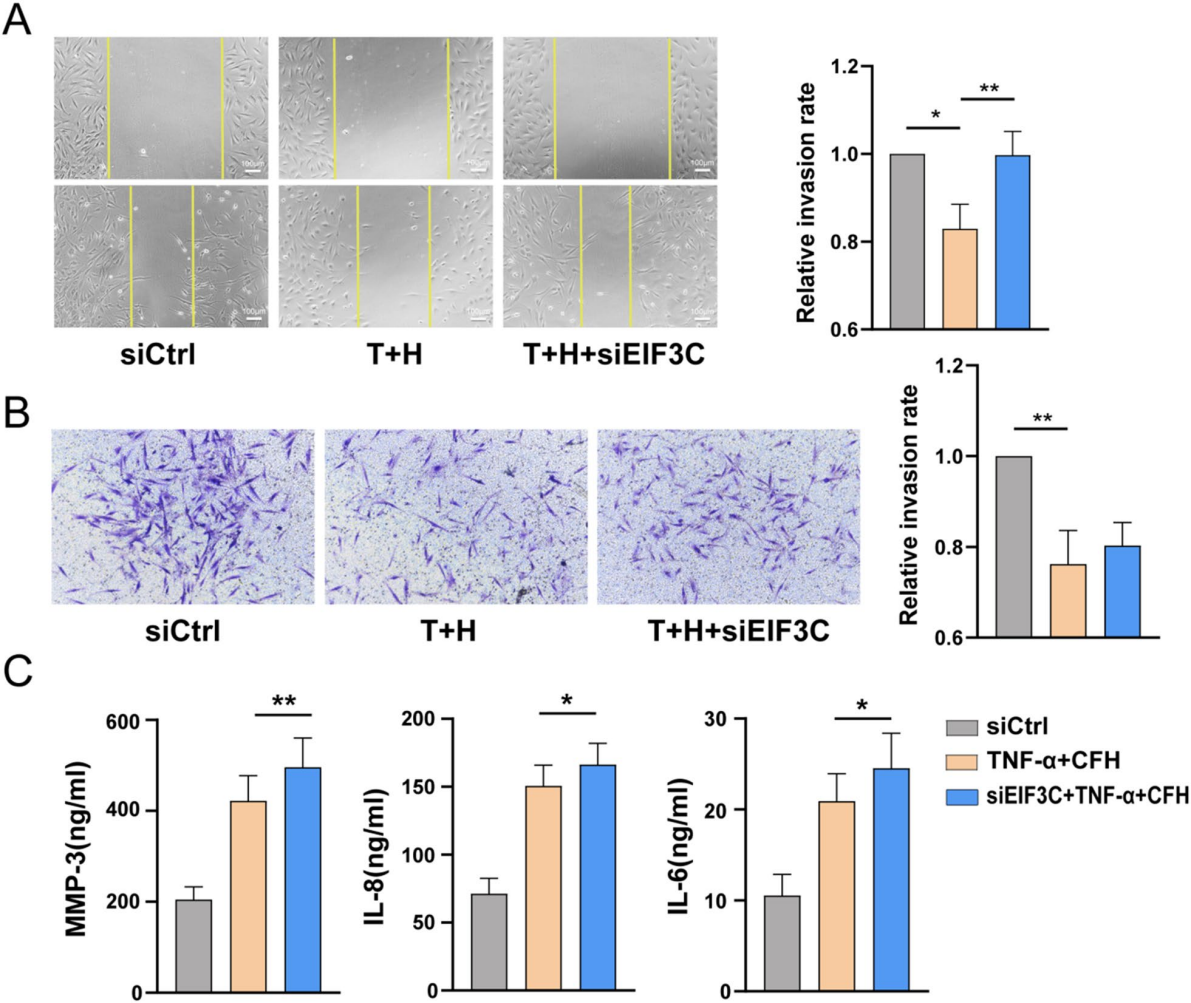
Effect of CFH knockdown on the function of RA FLS. RA FLSs were transfected with siRNAs for EIF3C (siEIF3C) or control siRNA (siCtrl) for 72 h and then stimulated with TNF-α(50 ng/ml) + CFH (5 μg/ml) for 24 h (n = 4–8). **A** Effect of EIF3C knockdown on the migration of RA FLSs was measured with the wound-healing assay. The relative migration rates were calculated by the percentage by which the original scratch area decreased and then normalized to that in the control group. **B** Effect of EIF3C knockdown on the invasion of RA FLSs using Transwell assay. The relative invasion rates were calculated by counting invaded cells and then normalized to that in the control group. Representative images (original magnification, × 200) are shown. **C** Effect of EIF3C knockdown on the inflammatory mediators of RA FLSs detected by ELISA. Data show the mean ± SEM. *p < 0.05; **p < 0.01

Finally, we used siRNA oligonucleotide sequences to downregulate the expression of EIF3C and further investigate the role of EIF3C in regulating RA FLS function. Both RT-qPCR and WB results showed that EIF3C-siRNA decreased the expression of CFH (Additional file 2: Fig. S3). As shown in Fig. 5A, the migration of RA FLSs was promoted upon EIF3C knockdown under the stimulation of CFH combined with TNF-α. However, there were no significant changes in the invasion of RA FLSs (Fig. 5B). In addition, we found that EIF3C knockdown also upregulated the expression of IL-6, IL-8, and MMP-3 (Fig. 5C). These data suggested that EIF3C is a potential target for CFH to play a role in inhibiting FLS function induced by TNF-α.

## Discussion

In this study, we have unfolded the anti-inflammatory roles of CFH in RA. We showed that CFH could inhibit the migration and invasion of FLS. Moreover, we found that CFH could dampen the inflammatory status of RA by inhibiting TNF-α-induced monocytes pyroptosis as well as inflammatory mediator production of monocytes and FLSs.

RA-FLS migration and invasion are pivotal contributors to synovitis and bone destruction. Many studies have shown that RA-FLS maintain their invasive, tumor-like phenotype despite previous passaging in vitro [17, 18]. A recent study demonstrated that synovial fibroblasts exhibited enhanced metabolic activity inducing functional changes with intensified migration, invasiveness, and osteoclastogenesis after repeated inflammatory challenges, which depended on intracellular complement C3 and C3a receptor expression [19]. CFH could accelerate the decay of the alternative pathway C3 convertase (C3b, Bb) [8]. Here, we found that CFH could directly inhibit RA-FLS migration and invasion. It would be an interesting topic to further explore the underline molecular mechanism and potential therapeutic targets to revert the phenotype of RA-FLS.

Our previous study has uncovered the role of complement C1q with its ligand PTX3 in promoting NLRP3 inflammasome over-activation, GSDMD-conferred pyroptosis, and inflammatory cytokine release in RA-derived monocytes [6]. A recent study by Zhai et al. supports the notion of a pathogenic role of GSDME [13]. Here, we have found that TNF-α could induce increased expression of GSDME-N, while CFH, in combination with TNF-α, significantly decreased the expression of cleaved caspase-3 and GSDME-N. Moreover, TNF-α could suppress CFH production in RA-derived monocytes, while this observation is the opposite in HC-derived monocytes. These suggest TNF-α exacerbates pyroptosis-conferred inflammation by inhibiting CFH production in RA patients. However, whether there is any interaction between the complement system and TNF-α is still a controversial issue [20]. TNF-α seems to activate the complement system, as demonstrated by a reduction of complement cleavage products in sera of patients treated with anti-TNF agents [21]. A mechanism proposed is that anti-TNFα decreases plasma levels of CRP, which can activate complement cascade through the classical pathway [22].

Our study also found that CFH significantly inhibited the secretion of TNF-induced inflammatory factors of local FLS in joints through upregulating EIF3C. EIF3C, one of the 13 subunits of eIF3 factor, is highly conserved in evolution and constitutes the function core of eIF3 with the other five subunits. EIF3C plays a very important role during development and homeostasis. Research on eIF3 is mostly concentrated in malignant tumors. EIF3C promotes the proliferation, migration, and invasion of prostate cancer, pancreatic cancer, and lung adenocarcinoma [23–25]. It has also been found that expression of EIF3C in HCC cells reduced trans-well cell migration [26]. However, knockdown EIF3C did not revert CFH-induced RA-FLS invasion significantly, indicating that the role of CFH was not dependent on EIF3C. Interestingly, the knockdown of EIF3C reversed the anti-inflammatory role of CFH in TNF-α-induced FLS inflammation. Recent studies suggest that the knockdown of EIF3C can increase the expression of caspase 3 to promote apoptosis [27]. Caspase 3 could convert TNF-α-induced apoptosis to cell pyroptosis and lead to inflammatory injury [28]. Therefore, it is hypothesized that CFH protects against the TNF-induced Casp-3-GSDME pyroptosis pathway by increasing the expression of EIF3C. Further studies are required to identify the related signaling pathways of CFH on RA and determine its relief effect on RA in vivo.

In this study, TNF-α did not promote FLS migration and invasion, which is also found in another study [29]. RA animal models demonstrated that TNF is not able on its own to promote synovial invasion and attachment to cartilage and bone. The study of hTNF transgenic mice showed that these processes were dependent on the presence of IL-1 or cartilage damage [30, 31].

IL-1 is a master cytokine of local and systemic inflammation. In patients failing TNFα blockers, IL-1 blockade is effective in controlling disease activity [32]. We found that IL-1β could significantly promote the expression of CFH in FLSs and healthy controls-derived monocytes. IL‐1/IL‐1R signaling induced by all‐trans‐retinal contributes to complement alternative pathway activation in retinal pigment epithelium [33]. Interaction of factor H with its receptor could stimulate the secretion of IL-1β by monocytes [34]. A miR-146a–CFH–IL-1β loop circuit was found to initiate a cascade of inflammation in temporal lobe epilepsy [35]. Therefore, we would further our research on whether CFH can also inhibit IL-1β-induced inflammation in RA patients.

In summary, this study shows that CFH levels may reflect the presence of an underlying inflammatory process in RA and plays a negative feedback-regulating role under TNF stimulation. CFH plays an important protective role in the joint via suppressing the migration and invasion of RA-FLS. Furthermore, CFH attenuates TNF-α-induced inflammation by upregulating EIF3C. Possible intervention strategies could be explored further to target CFH-related signaling pathways in anti-TNF-resistant RA patients.

### Supplementary Information

**Additional file 1:** A detailed list of the differentially expressed genes of RA-derived monocytes and FLS.

**Additional file 2: Figure S1**. A Monocytes were treated with IL-18, IL-17A, GM-CSF, and IL-10 (50 ng/ml) for 24 h. The expression of secreted CFH in the culture supernatant of RA patients was detected by ELISA. **B** FLS were treated with TNF-α, IL-1β, and IL-6 (50 ng/ml) for 24 h. The expression of CFH in RA-FLS and OA-FLS stimulated with TNF-α, IL-1β, and IL-6 was detected by qPCR. Data are expressed as mean ± SEM (n=6-8). *p < 0.05. **Figure S2.** Cells were treated with TNF-α（50ng/ml）or TNF-α（50 ng/ml）+CFH (5 μg/ml) or CFH (5 μg/ml) for 24 h. The CCK-8 assay was used to detect cell viability. Data are expressed as mean ± SEM (n=6). **Figure S3.** The efficiency of EIF3C knockdown was detected by RT-qPCR analysis and Western blotting. Data are expressed as mean ± SEM (n=5). **p < 0.01; ***p < 0.001. **Table S1.** Demographic and clinical features of included RA patients. **Table S2.** Demographic and clinical features of patients with OA. **Table S3.** Sequences of primers for RT-qPCR and siRNA oligonucleotides

## Data Availability

Data from RNA sequencing have been deposited in National Genomics Data Center (NGDC) and are accessible through the GSA-human Series accession number [HRA005914]. The rest of the data generated during this study is available within the article or its supplementary materials.
