# Supplementary material for: Complement factor H attenuates TNF-α-induced inflammation by upregulating EIF3C in rheumatoid arthritis
Source: J Transl Med. 2023 Nov 23;21:846. doi: 10.1186/s12967-023-04730-2 (PMC10668393; doi:10.1186/s12967-023-04730-2)
Supplement: Supplementary file 2 — Additional file 2: Figure S1. A Monocytes were treated with IL-18, IL-17A, GM-CSF, and IL-10 (50 ng/ml) for 24 h. The expression of secreted CFH in the culture supernatant of RA patients was detected by ELISA. B FLS were treated with TNF-α, IL-1β, and IL-6 (50 ng/ml) for 24 h. The expression of CFH in RA-FLS and OA-FLS stimulated with TNF-α, IL-1β, and IL-6 was detected by qPCR. Data are expressed as mean ± SEM (n=6-8). *p < 0.05. Figure S2. Cells were treated with TNF-α（50ng/ml）or TNF-α（50 ng/ml）+CFH (5 μg/ml) or CFH (5 μg/ml) for 24 h. The CCK-8 assay was used to detect cell viability. Data are expressed as mean ± SEM (n=6). Figure S3. The efficiency of EIF3C knockdown was detected by RT-qPCR analysis and Western blotting. Data are expressed as mean ± SEM (n=5). **p < 0.01; ***p < 0.001. Table S1. Demographic and clinical features of included RA patients. Table S2. Demographic and clinical features of patients with OA. Table S3. Sequences of primers for RT-qPCR and siRNA oligonucleotides [file 12967_2023_4730_MOESM2_ESM.pdf]

Supplementary Figure 1

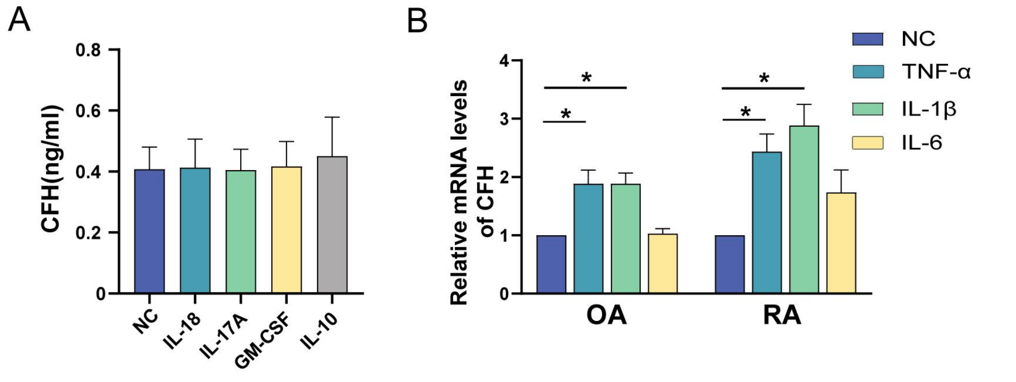

Supplementary Figure 2

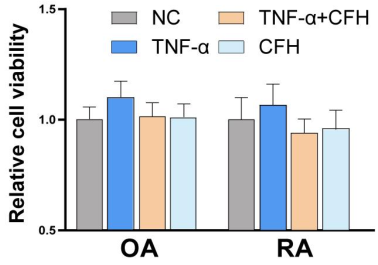

Supplementary Figure 3

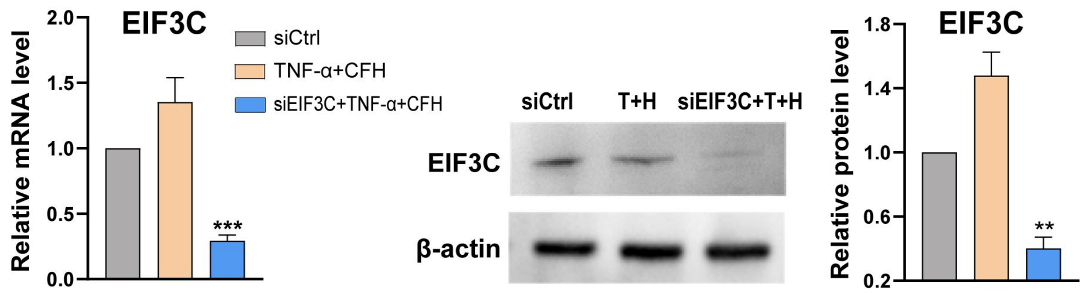

Fig. S1: **A** monocytes were treated with IL-18, IL-17A, GM-CSF, and IL-10 (50 ng/ml) for 24h.The expression of secreted CFH in the culture supernatant of RA patients was detected by ELISA. **B** FLS were treated with TNF-α, IL-1β, and IL-6 (50 ng/ml) for 24h. The expression of CFH in RA-FLS and OA-FLS stimulated with TNF-α, IL-1β, and IL-6 was detected by qPCR. Data are expressed as mean ± SEM (n=6-8). *p < 0.05.

Fig. S2: Cells were treated with TNF-α（50ng/ml）or TNF-α（50ng/ml）+CFH (5μg/ml) or CFH (5μg/ml) for 24 h. The CCK-8 assay was used to detect cell viability. Data are expressed as mean ± SEM (n=6).

Fig. S3: The efficiency of EIF3C knockdown was detected by RT-qPCR analysis and Western blotting. Data are expressed as mean ± SEM (n=5). **p < 0 .01; ***p < 0 .001.

Table S1. Demographic and clinical features of included RA patients

Age, yrs (mean ± SD)	51.39±13.72
Female: Male	59:13
Disease duration, yrs (mean±SD)	12.40±5.36
Rheumatoid factor-positive (%)	81
Anti-CCP-positive (%)	67
ESR	56±17.67
DAS28 (mean±SD)	5.2±0.9
Previous medications (%)	
Prednisone	42
Methotrexate	55
Leflunomide	37
Hydroxychloroquine	17
NSAIDs	35

Table S2. Demographic and clinical features of patients with OA

Age, yrs (mean ± SD)	71±8.5
Female: Male	6:8
Disease duration, yrs (mean±SD)	14.90±6.62
VAS	6.20±1.42

Table S3: Sequences of primers for RT-qPCR and siRNA oligonucleotides

gene		sequences
CFH	Forward	GTGAAGTGTTTACCAGTGACAGC
	Reverse	AACCGTACTGCTTGTCCAAAA
EIF3C	Forward	CAAGTTCAATATCATCGCCTCT
	Reverse	CATCCATTCGTTCCACCA
β-actin	Forward	CATGTACGTTGCTATCCAGGC
	Reverse	CTCCTTAATGTCACGCACGAT
EIF3C	siRNA	GCACACCTACTACAAGTTT
